# Violent and non-violent methods of attempted and completed suicide in Swedish young men: the role of early risk factors

**DOI:** 10.1186/s12888-015-0570-2

**Published:** 2015-08-14

**Authors:** Marlene Stenbacka, Jussi Jokinen

**Affiliations:** 1 Department of Clinical Neuroscience, Karolinska Institutet, Stockholm, Sweden; 2 Department of Clinical Sciences, Umeå University, Umeå, Sweden

**Keywords:** Suicide, Suicide attempt, Violent methods, Risk factors, Substance use

## Abstract

**Background:**

There is a paucity of studies on the role of early risk factors for the choice of methods for violent suicide attempts. Adolescent risk factors for the choice of violent or non-violent methods for suicide attempts and the risk of subsequent suicide were studied using a longitudinal design.

**Methods:**

A national Swedish cohort of 48 834 18–20-year-old young men conscripted for military service from 1969 to 1970 was followed through official registers during a 37-year period. Two questionnaires concerning their psychosocial background were answered by each conscript. Cox proportional hazard regression analyses were used to estimate the risk for different methods of attempted suicide and later suicide.

**Results:**

A total of 1195 (2.4 %) men had made a suicide attempt and of these, 133 (11.1 %) committed suicide later. The number of suicide victims among the non-attempters was 482 (1 %). Half of the suicides occurred during the same year as the attempt. Suicide victims had earlier onset of suicidal behaviour and had more often used hanging as a method of attempted suicide than those who did not later commit suicide. The early risk factors for both violent and non-violent methods of suicide attempt were quite similar.

**Conclusion:**

Violent suicide attempts, especially by hanging, are associated with a clearly elevated suicide risk in men and require special clinical and public health attention. The early risk factors related to the choice of either a violent or a non-violent suicide attempt method are interlinked and circumstantial factors temporally close to the suicide attempt, such as access to a specific method, may partly explain the choice of method.

## Background

Suicide is an important public health problem and one of the leading causes of death among young people worldwide. More than 1 million people die due to suicide per year and the rates seem to be similar in both developed and developing countries [1, 2]. In the Nordic countries, suicide was the second most common cause of death in the age category 15–24 years [3].

Furthermore, attempted suicide is up to 10–40 times more frequent than completed suicide and is considered to be the most predictive clinical risk factor for subsequent suicide. In a study from Finland, 8 % of suicide attempters committed suicide during a follow-up of 12 years [4]. A higher life-time risk of suicide of about 15 % after a suicide attempt in patients with major depression has been reported [5]. The risk of suicide seems to be highest during the first year following the index attempt [4, 5].

High suicide intent and violent suicide attempt methods are both related to an elevated suicide risk compared to suicide attempts with low intent or non-violent suicide attempt methods [6–8]. Having a disadvantaged background including low socio-economic status, parental psychiatric problems, i.e., high rates of depression and substance use, has been shown to be associated with suicide [9, 10]. Violent behaviour and criminality, especially violent offending, have also been pointed out as risk factors for suicide [11, 12]. In a study of Swedish conscripts focusing on psychiatric diagnoses at late adolescence, neurosis and personality disorders were strong predictors of both suicide attempt and suicide in the early and late phase of the follow-up period of 37 years [13, 14].

Suicide attempters using violent methods differ from non-violent attempters and may share more characteristics with suicide completers [15]. Even though there are distinct neurobiological and neuropsychological differences between violent and non-violent suicide attempters [16–19], there is a paucity of studies on the role of early risk factors for the choice of violent suicide attempt methods.

Knowledge about risk factors for a specific method of suicide attempt or suicide is important for prevention and for evaluation of suicide risk in clinical settings [7]. In this study, we were able to investigate the individual methods of attempted suicide, including both violent and non-violent methods.

The aim of this study was to analyse early risk factors for violent and non-violent methods of attempted suicide in a cohort of 48 834 conscripted men in 1969–70 in Sweden during a 37-year period. Another aim was to investigate methods of attempted suicide and the risk for later completed suicide.

The research questions were:

1. Are there specific early predictors of the choice of violent and non-violent methods for attempted suicide?
2. Do violent methods of attempted suicide lead more often to completed suicide when taking into account early confounders and co-morbid substance abuse?

## Methods

### Participants

This study is based on data from a longitudinal cohort of 48 834 young Swedish men conscripted for military service from 1 July 1969 to 30 June 1970. We included conscripts who were born from 1949 to1951 in order to get as homogeneous a cohort as possible. Six per cent of the conscripts were born in 1949, 17 % in 1950 and 75 % in 1951. In total, 48 834 out of 50 645 conscripts were included in the study. The mean age at the end of the follow-up was about 55–56 years for survivors.

### Measures

#### Measurement of potential predictors

All the participants were asked to fill in two non-anonymous voluntary questionnaires, the first of which included questions about family upbringing, psychosocial background and physical and psychological health and the second questions about substances used, including alcohol. The questionnaires have been found to have good validity and are therefore considered to be suitable for longitudinal studies [20, 21].

The predictors were selected from the questionnaire data, based on previous scientific studies and earlier studies of this cohort [22–24].

The selected predictors were:

Social class: Based on the father’s occupation, social class was assigned as: social class I + II vs social class III [25].

Medication for nervous problems among parents or other members in the family (yes, at least one parent or family member vs no).

Conduct problems at school (yes, at least once vs no).

Any prior contact with police and juvenile authorities (yes, at least once vs no).

Having taken medication for psychiatric disorder (yes, at least once vs no).

Two psychological variables were included: Emotional control and intellectual ability.

These were assessed by trained military psychologists who used both questionnaire and structured interview data. Emotional control was a measure of both mental and emotional stability and maturity, including tolerance to mental stress. The psychologist combined questionnaire and interview data to rate emotional control. The measures were assessed on a 5-point Likert scale ranging from (1) very low to (5) very high and expressed as (1–2 vs. 3–5). The ratings were regularly checked for interrater reliability, which was satisfactory [26]. If the psychologist found that the conscript suffered from any psychiatric disorder, a referral to a psychiatrist was issued and an eventual diagnosis was coded according to ICD-8. Details of this procedure and the validity of the assessments have been described earlier [14, 27].

Intelligence quotient (IQ) was based on four main intellectual and cognitive tests, measuring verbal, logical-inductive, spatial, and technical and mechanical ability [28, 29]. The scale ranged from 1 = very bad to 5 = very good, expressed as intellectual capacity (1–3 vs. 4–5) [28].

Smoking: (yes, more than 10 cigarettes per day vs less than 10 or none)

Problematic alcohol use was dichotomised (yes vs. no, with yes defined as ≥210 g pure alcohol per week, having ever taken an ‘eye-opener,’ being intoxicated often, having been taken into custody for public drunkenness on at least one occasion.

Drug misuse was coded (yes vs no), with yes defined as used illicit drugs 10 times or more or had taken drugs intravenously. Sniffing of solvents: (yes, once or more vs no) Table 1.

**Table 1:** Distribution of early risk factors measured at conscription in relation to suicide attempt and completed suicide. Percent

	Total cohort	Suicide attempt	Suicide
Fathers’ social class			
III	58.57	61.77	59.93
1-II	35.60	28.34	30.33
Missing	5.83	9.89	9.74
Medication for nervous problems in the family			
Yes (parents/ family members)	31.87	45.05	42.28
No	64.85	49.50	53.01
Missing	3.29	5.45	4.72
Fathers’ heavy drinking			
Often	4.04	9.06	6.67
Sometimes/occasionally never	93.27	86.83	89.27
Missing	2.69	4.11	4.07
Conduct problems at school			
Yes (at least once)	23.87	44.80	38.70
No	74.97	52.77	59.19
Missing	1.16	2.43	2.11
Own medication for psychiatric problems			
Yes	11.33	30.12	22.93
No	87.37	67.95	75.12
Missing	1.30	1.93	1.95
Emotional control			
Very bad, bad	30.21	53.36	47.80
Very good, good, medium	68.93	44.63	50.57
Missing	0.86	2.01	1.63
Intellectual ability			
Below average	19.04	34.73	30.57
Above average, average	80.81	64.51	69.27
Missing	0.15	0.76	0.16
Psychiatric diagnosis (at conscription)			
Yes (at least one diagnosis)	12.45	32.38	27.48
No	85.75	65.44	71.06
Missing	1.79	2.18	1.46
Contact with police and juvenile authorities			
Yes (several or some times	28.20	53.52	44.55
No	70.19	43.96	52.85
Missing	1.62	2.52	2.60
Risky alcohol use			
Yes	8.51	22.40	20.65
No	89.98	75.34	76.59
Missing	1.51	2.27	1.26
Dug misuse			
Yes	3.59	11.83	9.59
No	91.75	81.96	83.90
Missing	4.66	6.21	6.50
Sniffing			
At least once	13.26	28.10	23.41
No	85.05	68.88	73.33
missing	1.69	3.02	3.25
Hospitalization			
Alcohol hospitalization			
Yes (at least once)	5.78	25.4	25.20
No	94.22	74.59	74.80
Drug use hospitalization			
Yes (at least once)	1.37	19.46	12.03
No	98.63	80.54	87.97

### Registry data

Information on inpatient care was retrieved from the National Swedish Inpatient Register in order to identify inpatient care with an alcohol or drug diagnosis of dependence or abuse according to ICD-8 and ICD-9 from 1987 and ICD-10 from 1997 onwards.

The Register includes details concerning inpatient care stays and days at hospital and diagnoses. It was started in 1964 and since 1972 covers all public hospitals in Stockholm and Uppsala County, amounting to 85 % of all Swedish public inpatient care stays since 1983 and about 98–99 % since 1987.

The ICD classifications for hospitalisation and mortality were: Suicide attempt: ICD-8 and ICD- 9: Determined suicide attempt: E950-E959 and undetermined E980-E989: ICD-10: Determined X60-X84 and undetermined suicide attempt: Y10-Y34.

The National Board of Health and Welfare provides the Cause of Death Register containing mortality data which covers more than 99 % of all deaths occurring in Sweden from 1961 and onwards and is based on information from death certificates. Validity checks of death certificates are continuously performed, additionally; the majority of all suicide cases and other unnatural deaths are undergoing autopsy investigation [30]. Suicide attempts and suicides are often underreported or reported as "undetermined" causes. Therefore, determined and “undetermined” suicidal behaviour were combined in order to compensate for regional and temporal variation in ascertainment methods and to limit underreporting. Suicide was classified according to ICD-8 and ICD-9: E950-E959 and ICD-10: X60-X84 or as suicide with undetermined intent ICD-8 and ICD-9: E980-E989 and ICD-10: Y10-Y34.

Inpatient and mortality data was linked at Statistics Sweden via the unique civic registration number for each individual in the cohort. This number was then replaced with an individual serial number, making the data anonymous to the researchers, after approval by the Regional Ethical Review Board in Stockholm (Dnr 2007/174-31, Dnr 2008/1086-31/5). Using this procedure to guarantee the anonymity of data, the participants did not have to sign their informed consent.

### Missing data

Conscription in Sweden was mandatory during 1969–1970 for all Swedish male citizens who were not exempted for medical or psychiatric reasons. The questionnaires were completed by most conscripts, but some items in the questionnaires were not answered Table 1.

In the multivariate analyses we included only significant variables from the earlier bivariate analyses in relation to the outcomes. We included only those subjects who had answered all the questions in the multivariate models.

### Statistical analyses

Crude and multivariate Cox proportional regression analyses were used to calculate hazard ratios (HRs) with 95 % confidence intervals (95 % CI) for violent methods: hanging, shooting jumping from heights, moving train, cutting, drowning, other methods and non-violent suicide method: poisoning. If a subject had more than one suicide attempt, we defined the method of the first attempt as the index attempt during the observation period. We adjusted for confounders measured at conscription in relation to time to suicide. The surveillance time was calculated from September 1969 until death or the 1st January 2007 for all subjects in the cohort. Taking into account the surveillance time for the deceased subjects, the mean follow-up time was 36.2 years.

We had no exact information on whether a person had emigrated from Sweden or not during the follow-up period; therefore, we could not censor for emigration in the calculation of person time. To be able to compare the different methods for attempted suicide, we used the index attempt throughout the analyses.

Early predictors time were measured at the time of conscription and only once, for which reason the exact order in time was difficult to prove. Some of these variables such as alcohol or substance use could act as predictors or mediators of attempted and completed suicide.

The proportional hazard assumption was tested for each predictor (X) in crude and multivariate analyses by using a time-dependent explanatory variable in the model (X*(log time – average value of the log time). If the *P*-value was significant (*P < .*05) the proportional hazard assumption was not fulfilled and the variable was excluded from the analyses.

Kaplan-Meier survival curves were used to plot survival probability of suicide in participants with and without a history of attempted suicide during the follow-up. Survival analysis of suicide risk was calculated as the ratio of the number of suicides occurring during each time interval to the number of cases at risk i.e. that entered the respective interval alive. Time censoring: all participants were followed-up at least 36 years except for those who died earlier.

The analyses were performed using SAS Statistical Software 9.4 [31].

## Results

**Fig. 1:**
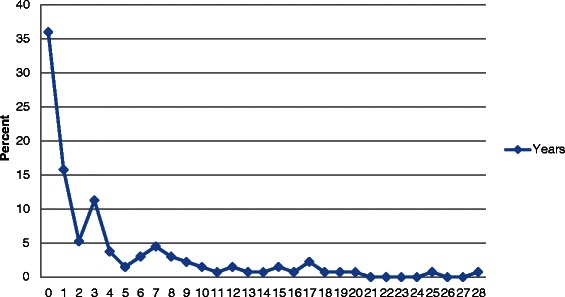
Time-lag between the first suicide attempt and subsequent suicide in 133 men, expressed in years. Percent

**Fig. 2:**
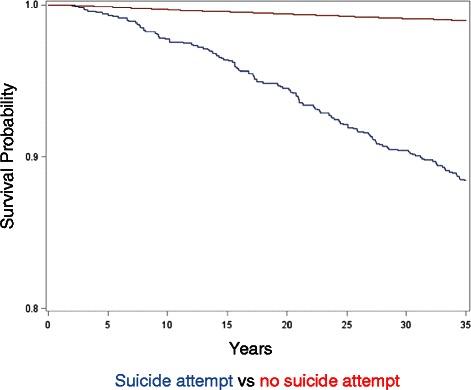
Survival curves for suicide in attempters and non-attempters

During the follow-up, 1195 men (2.4 %) had made a suicide attempt. Of these, 133 (11.1 %) later committed suicide. The number of suicide victims among the non-attempters was 482 (1 %). Most of the suicides occurred during the same year as the attempt (51.9 %) (Fig. 1). The survival of the suicide attempters was decreased by about 12 % and of non-attempters by 1 % (Fig. 2).

Methods used in all 1195 suicide attempts and suicides are presented in Table 2. Poisoning was the most usual method (77.9 %), followed by cutting or piercing (7.9 %). Attempted suicide by hanging was evident for 37 men (3.1 %) and jumping from a height for 25 men (2.1 %). The three most frequent suicide methods were poisoning (45.2 %); hanging/strangulation (24.3 %) and firearm use (11.2 %). Most of the methods of suicide completion were regarded as violent (n = 337, 55 %), while non-violent suicide methods were evident in 278 cases (45 %).

**Table 2:** Methods of attempted suicide and suicide in conscription cohort

Suicide methods	Index attempt	Suicide
	N = 1195	N = 615
	N (%)	N (%)
Non-violent method		
Poisoning	931(77.9)	278(45.2)
Violent methods		
Hanging, strangulation/suffocation	37(3.1)	149(24.3)
Drowning	8(0.7)	41(6.7)
Firearm	18(1.5)	69(11.2)
Cutting or piercing	94(7.9)	16(2.6)
Jumping from a height	25(2.1)	27(4.4)
Traffic	1(0.08)	22(3.6)
Other violent methods	81(6.8)	13(2.1)

### Early risk factors for a choice of suicide attempt method

An overview of proportions of all risk factors in suicide attempters, suicide victims and in the total cohort is presented in Table 1. The proportions were in general highest for suicide attempters followed by suicide victims and the entire cohort.

Table 3 shows multivariate Cox proportional analyses of risk factors for violent and non-violent methods of index suicide attempts compared to non-attempters. All risk factors included in the table were significantly associated with both non-violent and violent suicide attempt methods in the bivariate analyses (not shown in the table). Own medication for psychiatric problems, low intelligence, conduct problems at school, contact with police or juvenile authorities and sniffing solvents remained significant predictors of both violent and non-violent methods in multivariate analyses. Conduct problems at school yielded the highest hazard for the choice of violent method for a suicide attempt (HR = 2.16, 95 % CI = 1.62-2.89) followed by own medication for psychiatric problems (HR = 2.12, 95 % CI = 1.52-2.96) using non-attempter group as the reference group.

**Table 3:** Risk factors for violent and non-violent index suicide attempts in total cohort of 48834 Swedish young men

Variables	Violent vs. no suicide attempt ^a^Multivariate HR, 95 % CI	Non-violent vs. no suicide attempt ^b^Multivariate HR, 95 % CI
Family nervous problems (yes vs. no)	1.31, 0.99-1.72	1.18, 1.02-1.38
Fathers alcohol habits yes vs. no)	1.12, 0.68-1.84	1.33, 1.03-1.73
Own medication for psychiatric problems (no/yes)	2.12, 1.52-2.96	1.70, 1.40-2.05
Intelligence (Average or below average vs. above average )	1.75, 1.32-2.33	1.86, 1.60-2.18
Emotional control (low vs. high or medium )	1.24, 0.90-1.70	1.36, 1.14-1.63
Psychiatric diagnosis at conscription (yes vs. no)	1.06, 0.74-1.52	1.38, 1.14-1.68
Conduct problems at school (yes vs. no)	2.16, 1.62-2.89	1.43, 1.22-1.69
Contact with police or juvenile authorities (yes vs. no)	1.36, 1.0-1.82	1.80, 1.53-2.12
Smoking ≥10 cigarettes per day vs. <10 cigarettes per day	1.08, 0.81-1.44	1.26, 1.08-1.48
Problem drinking (yes vs. no)	1.31, 0.91-1.88	1.54, 1.27-1.87
Sniffing of solvents(yes vs. no)	1.58, 1.14-2.17	1.35, 1.13-162
Drug use (no vs. yes)	1.31, 0.82-2.08	1.20, 0.93-1.56

^a^Violent methods: hanging (*n* = 37), shooting (*n* = 18), jump from heights (*n* = 25), train move (*n* = 1), cutting (*n* = 94), drowning (*n* = 8), other methods (*n* = 81)

^b^Non-violent: poisoning (*n* = 931)

Multivariate Cox PH regressions. HR, 95 % CI

We also calculated the HRs for the same risk factors within the suicide attempter group, using violent suicide attempters as the exposed category and poisoning as the reference group. No specific early predictors for violent suicide attempt method were significant.

### Risk factors for suicide

Suicide victims had an earlier onset of suicidal behaviour. They were nearly three years younger at the time of the index attempt compared to surviving attempters (34.6 yrs, SD = 8.63 and (37.2 yrs, SD =8.99 years of age, respectively) (*P* = 0.0005). Furthermore, suicide victims had used violent methods, such as hanging, more frequently (*P* = 0.0095) and other (unidentified) methods (*P* = 0.028) less than those who did not committed suicide. Suicide attempt by hanging, strangulation or suffocation evinced a higher suicide risk in Model 1 (HR = 27.42, 95% CI, 14.18-53.03) and even after controlling for confounders measured at conscription (HR = 22.41, 95 % CI, 10.59–47.45) and additional alcohol and substance misuse (HR = 18.28, 95 %, CI 8.58-–38.97) (Table 4). In a fully adjusted model for poisoning as the method for attempted suicide, the risk for completed suicide was about four times lower (HR = 4.27, 95 % CI, 3.12-–5.84), compared with attempted suicide by hanging.

**Table 4:** Methods of attempted suicide as predictor of subsequent completed suicide

	Suicide		
	Model I^a^	Model II^b^	Model III^c^
Suicide attempt methods	HR (95 % CI)	HR (95 % CI)	HR (95 % CI)
Poisoning (yes vs no)	11.61, 9.38-14.38	7.17, 5.54-9.28	4.27, 3.12- 5.84
Hanging, strangulation or suffocation (yes vs no)	27.42, 14.18-53.03	22.41, 10.59- 47.45	18.28, 8.58- 38.97
Firearm (yes vs no)	11.72, 2.92-47.00	8.09, 2.00- 32.77	5.18, 1.27- 21.24
Cutting or piercing (yes vs no)	13.44, 7.58-23.82	8.86, 4.83- 16.24	5.74, 3.07- 10.73
Jumping from a height (yes vs no)	16.25, 6.07-43.48	12.88, 4.77- 34.84	8.74, 3.20-23.85
Other methods (yes vs no)	4.54, 1.70-12.34	3.44, 1.28-9.26	2.18,0.80-5.94

Multivariate Cox proportional regression analyses. HR, 95 % CI

^a^Multivariate model: including all methods in the model: poisoning, hanging, firearm, cutting, jumping and other methods.

^b^Adjusted for early confounders measurement at conscription (nervous problems in the family, social class, fathers’ alcohol habits, medication for own psychiatric problems, psychiatric diagnoses at conscription, intelligence, emotional control, conduct problems, sniffing, alcohol use, drug use)

^c^adjusted for early confounders measured at conscription (see above) and additional hospitalisation for alcohol and substance use

After stratifying for attempted suicide, 6 out of 13 early risk factors (low intellectual capacity, emotional control, psychiatric diagnose at conscription, conduct problems at school, earlier contacts with the police and alcohol use) had elevated HRs for suicide among non-attempters (not shown in tables).

## Discussion

In this long-term follow-up study of Swedish conscripts, we found that risk factors for the choice of violent and non-violent methods for attempted suicide were rather much the same. Conduct problems at school, low cognitive ability, and contact with police or juvenile authorities, psychiatric medication at conscription and sniffing solvents were predictors of both violent and non-violent methods of attempted suicide in multivariate analyses. Conduct problems at school showed the highest hazard for the choice of a violent method in multivariate analyses using non-attempters as a reference group, indicating that externalizing problems during childhood, earlier reported to be related to a higher suicide risk in males [32], may lead to the choice of a violent method, which in turn mediates a higher suicide risk. In a Finnish longitudinal population-based cohort study, a suicide outcome in form of completed suicide or a serious suicide attempt was predicted most strongly by co-morbid conduct and internalising problems in men [33]. Violent attempters have been suggested to be phenotypically more similar to suicide victims with clearly impaired decision-making and more low-grade neuroinflammation [17–19]. One earlier study on very early risk factors reported that short birth length for gestational age was more strongly related to violent than non-violent suicide attempts in men [34], whereas childhood trauma was not associated with the choice of violent methods in a study of suicide attempters [35]. However, childhood trauma has been associated with suicide attempts with higher intent [35, 36]. The relationship between suicide intent and the choice of violent methods is complex. Within the suicide attempter group, using violent suicide attempters as the exposed category and poisoning as the reference group, no early predictors for violent suicide attempt method were significant, indicating that early risk factors related to the choice of either a violent or a non-violent suicide attempt method are interlinked and that circumstantial factors temporally close to the suicide attempt, such as access to a specific method, may partly explain the choice of methods.

We found that the suicide risk was clearly elevated in suicide attempters and the first year after the attempt was a clear risk period, which is in line with the prevailing literature. National register and clinical studies on suicide attempters have demonstrated that attempted suicide is a strong risk factor for completed suicide. However, the majority of deaths by suicide occurred at the first attempt in this population-based cohort of male conscripts. Patients attempting suicide are a clinical high-risk group and an important target group for clinical suicide prevention, whereas the detection of individuals at risk for suicide not known at the clinic is a challenge for suicide prevention in the community. Suicide victims without prior suicide attempts shared the same early risk factors, such as low intellectual capacity, poor emotional control, psychiatric diagnoses at conscription, conduct problems at school, earlier contacts with the police and alcohol use, found also in those who survived the first suicide attempt. Haukka et al. [37] found in a large cohort of suicide attempters in Finland that the risk of completed suicide was 10 %; our finding of a suicide risk of 11 % after an attempted suicide is well in line with their results. Hanging as an attempted method of suicide was the most predictive of subsequent suicide in crude and multivariate models in our study. Those who had used hanging as an attempted method of suicide had a more than 20 times higher hazard to commit suicide. Even after controlling for treatment for alcohol or substance use and early confounders measured at the time of conscription, the hazard was still 18 times higher. Compared with suicide attempters by intoxication, suicide attempters by hanging had more than a fourfold higher risk for completed suicide. This finding is in line with the results of Runeson and co-workers, who reported a sixfold higher hazard of completed suicide after attempted suicide by hanging, using intoxication as a reference group [7]. However, in the present study, the number of persons who had used hanging as a method for attempted suicide attempt was rather small, which explains the wide confidence intervals in the analyses. Furthermore, most of those who had used hanging as a method died later by suicide and often with hanging as the method. Poisoning, the most frequently used method of suicide attempts, was associated with about a fourfold higher risk of dying by suicide in a fully adjusted model taking into account co-morbidity with substance abuse and early risk factors.

Interestingly, suicide victims had an earlier onset of suicidal behaviour than suicide attempters who survived during the follow-up. The age at first suicide attempt has been suggested to constitute a valid ‘candidate symptom’ for vulnerability factors for suicide [38]. Childhood adversity (particularly physical and sexual abuse) is associated with both an earlier onset of suicidal behavior [38] and completed suicide [11].

In agreement with other studies, we found that most of the completed suicide cases occurred close after the attempt [7], which should be noted by the staff who meet psychiatric patients and substance users, as well in order to prevent further attempts or suicide. Our findings indicate a need for detection of early risk factors in young adults and an intensive clinical follow-up of suicide attempters who use violent methods.

### Advantages and limitations

This study is based on a large national cohort using a longitudinal design and with information on several adolescent predictors, which is a clear advantage. Very few (2–3 %) of the conscripts were excluded from the study mainly because of a physical or psychiatric handicap. Non-anonymous questionnaires were used at conscription, which could have led to a lower response rate for especially problematic issues such as alcohol and substance use and other psychological and behavioural matters. Previous studies on the conscription cohort have compared register and self-reported information, for example, alcohol and substance use, and found good agreement between the different sources of data [27]. A small number of the conscripts did not answer to one or more of the items in the questionnaires. One could assume that these subjects had higher rates of substance use and other maladjusted behaviour measured at conscription, which in turn could have affected the outcome. However, we have no reason to believe that the risk to the outcome, violent and non-violent suicide attempts and suicide, has been considerably affected owing to the internal missing data. Suicide attempts preceding conscription could not be ascertained and we did not have information about all suicide attempters in the beginning of the study period, since the Inpatient Register was not complete before the year 1987. Thus the data may not extend to younger suicides which is a limitation.

Some subjects might have committed many attempts at suicide, especially in combination with substance misuse, and never been registered for a suicide attempt, which is a challenge for professionals whose task it is to identify individuals at high risk of suicide. Self-reported suicide attempts appear to be rather common, especially among drug users [39]. Furthermore, information on early adversity in the form of abuse was lacking.

## Conclusions

In conclusion, this study shows that the risk factors were much the same for both violent and non-violent methods of attempted suicide, indicating interlinked, complex pathomechanisms. Suicide attempters using violent methods have a very high completed suicide risk.

## Abbreviations

ICD: International statistical classification of diseases and related health problems
IQ: Intelligence quotient
HR: Hazard ratio
CI: Confidence interval
SD: Standard deviation

## References

[1] Bailey RK, Patel TC, Avenido J, Patel M, Jaleel M, Barker NC (2011). Suicide: current trends. J Natl Med Assoc.

[2] Borges G, Nock MK, Haro Abad JM, Hwang I, Sampson NA, Alonso J (2010). Twelve-month prevalence of and risk factors for suicide attempts in the World Health Organization World Mental Health Surveys. J Clin Psychiatry.

[3] Titelman D, Oskarsson H, Wahlbeck K, Nordentoft M, Mehlum L, Jiang GX (2013). Suicide mortality trends in the Nordic countries 1980–2009. Nord J Psychiatry.

[4] Suominen K, Isometsä ÄE, Ostamo A, Lönnqvist J (2004). Level of suicidal intent predicts overall mortality and suicide after attempted suicide: a 12-year follow-up study. BMC Psychiatry.

[5] Nordström P, Asberg M, Aberg-Wistedt A, Nordin C (1995). Attempted suicide predicts suicide risk in mood disorders. Acta Psychiatr Scand.

[6] Niméus A, Alsén M, Träskman-Bendz L (2002). High suicidal intent scores indicate future suicide. Arch Suicide Res.

[7] Runeson B, Tidemalm D, Dahlin M, Lichtenstein P, Långström N (2010). Method of attempted suicide as predictor of subsequent successful suicide: national long-term cohort study. BMJ.

[8] Stefansson J, Nordström P, Jokinen J (2012). Suicide Intent Scale in the prediction of suicide. J Affect Disord.

[9] Suokas J, Suominen K, Isometsä E, Ostamo A, Lönnqvist J (2001). Long-term risk factors for suicide mortality after attempted suicide – findings of a 14-year follow-up study. Acta Psychiatr Scand.

[10] Harris EC, Barraclough B (1997). Suicide as an outcome for mental disorders. A meta-analysis. Br J Psychiatr.

[11] Jokinen J, Forslund K, Ahnemarke E, Gustavsson JP, Nordström P, Karolinska AM (2010). Interpersonal Violence Scale predicts suicide in suicide attempters. J Clin Psychiatry.

[12] Stenbacka M, Romelsjö A, Jokinen J (2014). Criminality and suicide: a longitudinal Swedish cohort study. BMJ Open.

[13] Lundin A, Lundberg I, Allebeck P, Hemmingsson T (2011). Psychiatric diagnosis in late adolescence and long-term risk of suicide and suicide attempt. Acta Psychiatr Scand.

[14] Allebeck P, Allgulander C, Fisher LD (1988). Predictors of completed suicide in a cohort of 50 465 young men: role of personality and deviant behaviour. BMJ.

[15] Giner L, Jaussent I, Olié E, Béziat S, Guillaume S, Baca-Garcia E (2014). Violent and serious suicide attempters: one step closer to suicide?. J Clin Psychiatry.

[16] Jokinen J, Nordström AL, Nordström P (2010). Cholesterol, CSF 5-HIAA, violence and intent in suicidal men. Psychiatry Res.

[17] Jollant F, Bellivier F, Leboyer M, Astruc B, Torres S, Verdier R (2005). Impaired decision making in suicide attempters. Am J Psychiatry.

[18] Lindqvist D, Janelidze S, Erhardt S, Träskman-Bendzl L, Engström G, Brundin L (2011). CSF biomarkers in suicide attempters -- a principal component analysis. Acta Psychiatr Scand.

[19] Isung J, Aeinehband S, Mobarrez Fobarrez F, Nordström P, Runeson B. Interleukin-6 and impulsivity: determining the role of endophenotypes in attempted suicide. Transl Psychiatry. 2014;4. doi:10.1038/tp.2014.113. 10.1038/tp.2014.113 PMC4350519 25335166

[20] Andreasson S, Allebeck P, Romelsjo A (1988). Alcohol and mortality among young men: longitudinal study of Swedish conscripts. Br Med J.

[21] Eriksson A, Romelsjö A, Stenbacka M, Tengström A (2011). Early risk factors for criminal offending in schizophrenia: a 35-year longitudinal cohort study. Soc Psychiatr Epidemiol.

[22] Stenbacka M, Moberg T, Romelsjö A, Jokinen J (2012). Mortality and causes of death among violent offenders and victims – a Swedish population-based longitudinal study. BMC Public Health.

[23] Davstad I, Allebeck P, Leifman A, Romelsjö A (2011). Self-reported drug use and mortality among a nationwide sample of Swedish conscripts – A 35-year follow-up. Drug Alc Depend.

[24] Uppmark M, Karlsson G, Romelsjö A (1999). Drink driving and criminal behaviours as risk factors for receipt of disability pension and sick leave: a prospective study of young men. Addiction.

[25] Otto U (1976). Male youths. A sociopsychiatric study of a total annual population of Swedish adolescent boys. Acta Psychiatr Scand.

[26] Benson G, Holmberg MB (1985). Validity of questionnaire in population studies on drug use. Acta Psychiatr Scand.

[27] Rydelius P-A (1983). Alcohol-abusing teenage boys. Acta Psychiatr Scand.

[28] Ståhlberg B (1971). I-provet (Intelligence test: the Swedish enlistment battery).

[29] David AS, Malmberg L, Brandt L, Lewis G (1997). IQ and risk for schizophrenia: a population-based cohort study. Psychol Med.

[30] National Board of Health and Welfare (2012) The Cause of Death register. Stockholm, Sweden: Socialstyrelsen. 2013–8–6.

[31] SAS Institute Inc (2014). SAS® 9.4.

[32] Geoffroy M-C, Gunnell D, Power C (2014). Prenatal and childhood antecedents of suicide: 50-year follow-up of the 1958 British Birth Cohort study. Psychol Med.

[33] Sourander A, Klomek AB, Niemelä S, Haavisto A, Gyllenberg D, Helenius H (2009). Childhood predictors of completed and severe suicide attempts: findings from the Finnish 1981 Birth Cohort Study. Arch Gen Psychiatry.

[34] Mittendorfer-Rutze E, Wasserman D, Rasmussen F (2008). Fatal and childhood growth and the risk of violent and non-violent suicide attempts: a cohort study of 318,953 men. J Epidemiol Community Health.

[35] Lopez-Castroman J, Jaussent I, Beziat S, Guillaume S, Baca-Garcia E, Olié E (2014). Posttraumatic stress disorder following childhood abuse increases the severity of suicide attempts. J Affect Disord.

[36] Rajalin M, Hirvikoski T, Jokinen J (2013). Family history of suicide and exposure to interpersonal violence in childhood predict suicide in male suicide attempters. J Affect Disord.

[37] Haukka J, Suominen K, Partonen T, Lönnqvist J (2008). Determinants and outcomes of serious attempted suicide: a nationwide study in Finland, 1996–2003. Am J Epidemiol.

[38] Slama F, Courtet P, Golmard JL, Mathieu F, Guillaume S, Yon L (2009). Admixture analysis of age at first suicide attempt. J Psychiatr Res.

[39] Onyeka IN, Beynon CM, Uosukainen H, Korhonen MJ, Ilomäki J, Bell JS (2013). Coexisting social conditions and health problems among clients seeking treatment for illicit drug use in Finland: The HUUTI study. BMC Public Health.

